# Developing clinical practice guidelines: reviewing, reporting, and publishing guidelines; updating guidelines; and the emerging issues of enhancing guideline implementability and accounting for comorbid conditions in guideline development

**DOI:** 10.1186/1748-5908-7-62

**Published:** 2012-07-04

**Authors:** Paul Shekelle, Steven Woolf, Jeremy M Grimshaw, Holger J Schünemann, Martin P Eccles

**Affiliations:** 1 RAND Corporation, Santa Monica, CA 90407, USA; 2 Veterans Affairs Greater Los Angeles Healthcare System, Los Angeles, CA 90073, USA; 3 Department of Family Medicine and Center on Human Needs, Virginia Commonwealth University, Richmond, VA, USA; 4 Clinical Epidemiology Program, Ottawa Hospital Research Institute, Ottawa, ON, Canada; 5 Department of Medicine, University of Ottawa, Ottawa, ON, Canada; 6 Departments of Clinical Epidemiology and Biostatistics and of Medicine, McMaster University, Hamilton, Canada; 7 Institute of Health and Society, Newcastle University, Baddiley-Clark Building, Richardson Road, Newcastle upon Tyne, NE2 4AX, UK

## Abstract

Clinical practice guidelines are one of the foundations of efforts to improve health care. In 1999, we authored a paper about methods to develop guidelines. Since it was published, the methods of guideline development have progressed both in terms of methods and necessary procedures and the context for guideline development has changed with the emergence of guideline clearing houses and large scale guideline production organisations (such as the UK National Institute for Health and Clinical Excellence). It therefore seems timely to, in a series of three articles, update and extend our earlier paper. In this third paper we discuss the issues of: reviewing, reporting, and publishing guidelines; updating guidelines; and the two emerging issues of enhancing guideline implementability and how guideline developers should approach dealing with the issue of patients who will be the subject of guidelines having co-morbid conditions.

## Background

Clinical practice guidelines (hereafter referred to as guidelines) are one of the foundations for efforts to improve health care. The modern age of guidelines began with a 1992 IOM report, which defined guidelines as “systematically developed statements to assist practitioner and patient decisions about appropriate health care for specific clinical circumstances” [1]. In 1999, we authored a paper about methods to develop guidelines [2]. It covered: Identifying and refining the subject area of the guideline; Running guideline development groups; Identifying and assessing the evidence; Translating evidence into a clinical practice guideline; and Reviewing and updating guidelines. Since it was published, the methods of guideline development have progressed both in terms of methods and necessary procedures and the broad context for clinical practice guidelines has changed.

To help users identify and choose guidelines there has been the emergence of guideline clearing houses (such as the AHRQ Guideline Clearing House ( http://www.guideline.gov)) that identify and systematically characterize guidelines on a number of domains and the development of robust guideline appraisal instruments such as the AGREE tool [3,4]. There has been the appearance of large scale guideline production organisations both at a national level (such as the UK National Institute for Health and Clinical Excellence or Scottish Intercollegiate Guidelines Network) and a condition level (such as the Ontario Cancer Guideline Program). There have also been relevant reports (that some of us have participated in) for the World Health Organisation [5] and professional societies (Schünemann HJ, Woodhead M, Anzueto A, Buist AS, MacNee W, Rabe KF, Heffner J. A guide for guidelines for professional societies and other developers of recommendations: an official American Thoracic Society (ATS)/European Respiratory Society (ERS) Workshop Report; forthcoming). Such organizations and those interested in producing and using guidelines now have a high profile society in the Guidelines International Network ( http://www.g-i-n.net/). Against this background it seems timely to, in a series of three articles, update and extend our earlier paper on the methods of developing clinical practice guidelines. This series is based on a background paper [6] we prepared for the Institute of Medicine report “*Clinical Practice Guidelines We Can Trust*” [7].

In the first paper we discussed target audience(s) for guidelines, identifying topics for guidelines, guideline group composition and the processes by which guideline groups function and the important procedural issue of conflicts of interest. In the second paper we discussed issues of identifying and synthesizing evidence: deciding what type of evidence and outcomes to include in guidelines; integrating values into a guideline; incorporating economic considerations; synthesis, grading, presentation of evidence; and moving from evidence to recommendations. In this third and final paper in the series we discuss the issues of: reviewing, reporting, and publishing guidelines; updating guidelines; and two emerging issues - enhancing guideline implementability and how guideline developers should approach dealing with the issue of patients who will be the subject of guidelines having co-morbid conditions.

## Reviewing, reporting, and publishing guidelines

### Peer review and consultation

Only so many experts and perspectives can be represented on a guideline group. The review process provides an opportunity to elicit input from broader and important perspectives that the group itself cannot encompass but that have important insights into the evidence (and its contestability) or the challenges of adopting recommendations. This can be divided into the two steps of invited peer review, where the reviewers are identified by the guideline developers on the basis of their perceived ability to contribute, and public consultation, where the document is open to comment from any interested party.

#### Invited peer review

The means by which review comments are solicited is typically by mailing draft documents to the reviewers along with instructions on relevant criteria and a deadline for feedback. However, some groups obtain similar input and criticism earlier in the process by forming advisory groups that include some of the above stakeholders. The advisory group may not have voting power but may receive draft documents, correspondence, and updates as the guideline group deliberates. The group’s meetings can also be attended by observers who represent the above interest groups, or they may be invited to an open forum, hearing, or workshop to present papers and testimonials to the guideline group. These observers may share criticisms in person at such meetings or forward concerns offline in correspondence with the group or its organizational/agency sponsor. As an endeavor in scholarship, documents and recommendations developed by a guideline group should be critically reviewed by colleagues and experts to identify errors and omissions, suggestions for improvement and clarification, and debate about the rationale. The criticism is helpful in four ways (Table 1).

**Table 1:** Advantages of external review of a guideline

(1) Checking the accuracy, comprehensiveness, and balance of the scientific evidence
(2) Checking the validity of the rationale for recommendations
(3) Feedback on the clarity and feasibility of recommendations
(4) Engagement of stakeholders

Reviewers with expertise on relevant past research and studies in progress and mastery of the clinical topic can identify relevant studies that were overlooked, mistakes made in describing studies or their results, flaws in commentaries about the quality of the studies, methodological and statistical errors in pooling data in meta-analyses or models, and imbalance and asymmetry in presenting the evidence. Reviewers can challenge the logic used by the guideline group in translating the evidence into recommendations. They can note, for example, when the evidence presented does not support the recommendation offered and call attention to biases, political pressure, or other factors that may be coloring the group’s judgments. Reviewers with knowledge of the realities of clinical practice and the administration of health systems can provide useful feedback on how easily the recommendations can be adopted, or even understood, by clinicians and systems of care. Language issues that create ambiguities in explicating which patients are affected and the details of the intervention can be identified. Reviewers grounded in the “real world” can also identify broader policy ramifications, such as the lack of providers or technology to support a recommendation, implications for reimbursement or medico-legal liability, impracticalities created for information systems or performance review criteria, upcoming legislation or policies that bear on the topic, and political resistance the recommendations will face.

The group’s procedures for selecting reviewers should pay attention to this consideration in choosing potentially harsh critics who can articulate their scientific or clinical reasoning. It should also take note of the need to be explicit about conflicts of interest (as discussed in the first paper in this series). Inviting criticism from provider groups and specialty societies who are expected to be critical is also important.

#### Public consultation

It is prudent to share drafts with a spectrum of reviewers who include not only supporters but also the individuals, specialty groups, and industries expected to be critical of the group’s characterization of the evidence or of their recommendations. Reviewers can be given clear instructions to accompany their criticisms with relevant scientific evidence and citations to support their criticisms and suggestions. Reviewers who express strong views but cannot present a cogent scientific rationale may be less helpful than critics who can articulate a sound scientific and clinical argument for their views.

Whether or not their feedback is useful on substance, inviting comments and criticism from stakeholders is important for “buy-in” to give the major players a sense of partnership in the process rather than feeling excluded. Thus, even if scientific and clinical review is already provided by leading experts, comments are often invited from relevant specialty societies (e.g., American Academy of Pediatrics), disease-related organizations (e.g., American Diabetes Association), government agencies (e.g., Centers for Disease Control), and other bodies that are relevant to the topic in question. Perhaps the most useful benefit of review by stakeholders lies in potentially engaging their assistance in promoting awareness and adoption of the recommendations when they are released. Specialty societies and other entities that have played a role in reviewing and improving guidelines are often willing to participate in an outreach effort to promote its implementation.

#### Dealing with peer review

Inviting criticism of draft documents hardly guarantees that the final guideline will escape criticism. In particular, critics of guidelines can fault the review process if there is a perception that their criticisms were submitted but ignored. Therefore, it is useful for the guideline group to adopt an open, systematic and fair process for receiving and responding to review comments. For example, the group can maintain a database in which it documents every criticism or comment it receives from every reviewer, how the guideline was or was not modified, and the rationale for actions taken or for inaction. Making such a document available to the public is important for transparency, and some groups even post it on their website.

How groups react to review comments is, or ideally should be, consistent with the methodology it used in making the original recommendations. For example, if a group accepted expert opinion as a determinant of recommendations, criticism by experts that the recommendation is inappropriate or public or political disfavor might be reason enough for rewording the recommendation. Conversely, on the other extreme, if a group’s methodology insists on evidence from well-designed randomized controlled trials, neither strident expert opinions nor a lengthy bibliography of supporting cohort studies are necessarily grounds for revising the recommendations. The harshest criticism from a topic expert or organization might merit no action if the reviewer cannot produce evidence or rationale that is relevant to the group’s methodology for making recommendations. The group must hold an explicit methodology with explicit rules of evidence and criteria for recommendations, however, to justifiably invoke the latter as an excuse for ignoring criticisms.

### Peer-reviewed publications

Publishing practice guidelines (or the evidence reviews on which they are based) in a peer-reviewed publication offers an additional opportunity for critical appraisal by anonymous reviewers and feedback to improve the guidelines. Publication in a peer-reviewed journal also enhances the scientific credentials of the guideline and act as a “stamp of approval,” which can be important to some clinicians, patients, academia, and policymakers. For guideline groups and sponsoring organizations that lack the resources to widely publicize or disseminate guidelines on their own, journal publication provides a venue for distributing print copies and, increasingly, an online platform for full-text retrieval of electronic copies of the guidelines, evidence reviews, data tables, and other supplementary information.

Submitting guidelines for publication in a peer-reviewed journal is not without its disadvantages, however. First, the peer-review process, the need to revise and resubmit the guidelines, and rejections that require submission to other journals are time-consuming steps that can introduce lengthy delays in the release of guidelines. Once accepted, journals may require additional time for production of the issue and exercise partial or exclusive control over the timing of the release. A guideline may be published a year or more after its completion by the group, long after the publication date of the most recent studies reviewed by the group. By then, new studies or new interventions may make the guideline obsolete or at least dated. Second, editors may insist on modifications to the guideline or evidence review that are responsive to the comments of their reviewers but that change documents and language that the guideline group has carefully crafted, often in response to extensive review by leading authorities. The wording and structure recommended by this extensive process may run up against the recommendations of a handful of reviewers selected by the journal as part of its ordinary peer review process. Some journals address this problem by accepting a group’s review process in lieu of the journal’s normal review procedures. Third, either because of the above difficulties or the style and formatting policies of the journal, the peer-reviewed version may differ from other versions released by the guideline group or its sponsoring organization or agency, creating troubling inconsistencies.

### Other publications

Guideline groups now often develop multiple products to cater to a multiplicity of target audiences. For example, a short guideline summary may be intended for busy clinicians or policymakers whose interest lies only in knowing the core recommendation and not the underlying evidence or the group’s rationale. A technical report, which may include detailed evidence reviews, evidence summary tables, and modeling results, may be produced for audiences interested in studying the science and critiquing the quality of the scientific reasoning. A lay version of the guideline may be produced to explain the aspects of the guideline that are relevant to patients, often using language that is free of technical jargon and is accompanied by useful graphics or illustrations. The above documents are typically disseminated in an electronic format, as HTML or PDF documents accessible online, but some guidelines may also include a print publication in a peer-reviewed medical journal, to reach audiences who prefer print media or view the latter as an important metric for scientific credibility.

Two decades ago, whether as peer-reviewed or other publications, guidelines and related products (e.g., evidence reviews, data tables, modeling studies, patient education materials) were almost exclusively print publications that arrived in the mail and sat on shelves and desks. In the modern era, although print publications remain a staple, an increasing proportion of publications are available and more widely used electronically in searchable online HTML and PDF formats. Increasingly, guidelines are being provided with interactive “front ends” that allow users to navigate or interrogate the guideline or supporting evidence. Some guidelines or supporting materials are available for full-text retrieval at no charge and are therefore more accessible to the general public, whereas others require some payment for controlled access. Guidelines sometimes spawn related decision support tools or software to implement the guideline in practice.

## Updating guidelines

It is well recognized that guidelines become out of date and therefore require updating. We have previously argued that there were six situations (Table 2) that might necessitate the updating of a clinical practice guideline [8]. Changes in the values placed on outcomes often reflect societal norms. Measuring the values placed on outcomes and how these change over time is complex and has not been systematically studied. When changes occur in the availability of resources for health care or the costs of interventions, a generic policy on updating is unlikely to be helpful, because policy makers in disparate health care systems consider different factors in deciding whether services remain affordable. Most effort has been directed towards defining when new information on interventions, outcomes and performance justifies updating guidelines. This process includes two stages: 1) identifying significant new evidence, and 2) assessing whether the new evidence warrants updating. Within any individual guideline it is possible that there will be some recommendations that are invalid whilst others remain current. A guideline on congestive heart failure [9] for example, includes 37 individual recommendations. How many must be invalid to require updating the whole guideline? Clearly a guideline needs updating if the majority of recommendations are out-of-date, with new evidence demonstrating that the recommended interventions are inappropriate, ineffective, or superseded by new interventions. In other cases a single, outdated recommendation could invalidate the entire document. Judgments about whether a guideline needs updating are inherently subjective and reflect the clinical importance and number of invalid recommendations. Shekelle and colleagues described an operational method based on their conceptual model, presented above, in relation to the need for updating of 17 clinical guidelines published by the Agency for Healthcare Research and Quality [10]. They found that seven guidelines were so far out of date that a major update was required, an additional six guidelines required a minor update, three guidelines were still valid, and for one guideline they could reach no conclusion. They concluded that as a general rule, guidelines should be re-evaluated no less frequently than every three years. In an evaluation of the need for updating systematic reviews, Shojania and colleagues found that almost one quarter of systematic reviews are likely out-of-date at two years post-publication [11]. This method provided a way of balancing the resources required for updating with the potential benefits assuming that a full re-development of a guideline on each occasion was not necessarily an efficient use of resources. Gartlerhner and colleagues [12] explicitly addressed this issue when they compared this method (which they termed the review method) with a “traditional” method of updating (comparable to de novo guideline development) across six topics from the 1996 US Preventive Services Taskforce Guide to Clinical Preventive Services in terms of the completeness of study identification, the importance of any studies that were missed by either method and the effort involved in the methods. Their results showed that “Although the review approach identified fewer eligible studies than the traditional approach, none of the studies missed was rated as important by task force members acting as liaisons to the project with respect to whether the topic required an update. On average, the review approach produced substantially fewer citations to review than the traditional approach. The effort involved and potential time saving depended largely on the scope of the topic”. On the basis of this they concluded that ^“^The revised review approach provides an efficient and acceptable method for judging whether a guideline requires updating”.

**Table 2:** Situations that might necessitate the updating of a clinical practice guideline

1. Changes in the evidence on the existing benefits and harms of interventions:
2. Changes in the outcomes considered important:
3. Changes in the available interventions:
4. Changes in the evidence that current practice is optimal:
5. Changes in the values placed on outcomes:
6. Changes in the resources available for health care:

A description of updating in a cancer guidance program [13] concluded that for their purposes that a more frequent, three monthly, literature search was needed though they found that the productiveness of this varied across different guidelines.

These issues have been enshrined within the processes of some guideline development programs and in the UK the National Institute for Health and Clinical Excellence (NICE) recommends a combination of literature searching and professional opinion to inform the need for a “full” or “partial” updates and describes its processes for these - though they involve both changes in the evidence relating to a guideline and extensions of the scope of a guideline (changes in the outcomes considered important or available interventions). The assessment of the need for an update happens every three years. In the National Guideline Clearinghouse, guidelines are required to have been re-examined every three years.

## Emerging issues

There are two emerging issues around which there is considerably less evidence or experience to guide guideline developers - what guideline developers can do to enhance guideline implementability and how guideline developers should deal with the fact that, despite guidelines addressing single condition guidelines, many patients do not just have single clinical conditions. Strategies to promote the implementation of guidelines are beyond the scope of this paper but the interested reader is directed to the work of the Cochrane Collaboration Effective Practice and Organisation of Care Review Group in the Cochrane Library ( http://www.thecochranelibrary.com).

### Enhancing guideline implementability

Guidelines are not self implementing. Developing guidelines and making them available to health care professionals does not ensure their use. Whilst guideline developers may have some responsibility for guideline dissemination they rarely have responsibility for guideline implementation. Guideline dissemination is sometimes undertaken by the developer and sometimes undertaken by practitioners or managers of the health care system or professional societies or the pharmaceutical industry. By contrast, guideline implementation is usually the responsibility of practitioners or managers. As a result, it is not always clear what role guideline developers should have in dissemination and implementation.

There are studies that suggest that the specificity of guideline recommendations affects their use [14] and observational studies that suggest that attributes of recommendations affect clinicians report of how they would use them [15]. There is also an instrument for assessing implementability [16] and a recent article has proposed an implementability framework [17]. The proposed implementablity framework (Table 3) was developed by the authors and then tested against a number of guidelines. They reported that many of the guidelines did not include features that (even on current limited knowledge) are known to promote guideline use. However, they also acknowledge that it is an empirical question as to whether including such information increases guideline use. Apart from studies such as these, there is relatively little research evidence about how guideline developers should improve implementability but there are a number of ongoing studies that should provide guidance for developers in the next few years. If guideline developers wish to enhance the implementability of their guidelines then current best practice suggests that developers should develop formal relationships with those in health care systems responsible for guideline dissemination and implementation and then use strategies to support guideline uptake such as those in Table 3.

**Table 3:** Strategies to support guideline uptake

· Pre-emptive identification of potential barriers of recommendations, and a priori generation of solutions to address them by the guideline development group. At a minimum the guideline group should be aware of the potential barriers;
· Use of behaviorally specific language in the guideline [18][19][14];
· Use of multiple formats and channels for guideline dissemination based on preferences of the target group of health care practitioners;
· Development of educational resources adapted in content, and vehicle to each target group of health care practitioners;
· Identification of the resource implications of recommendations, ensuring their availability before starting;
· Use of data collection tools (for example, simple audit templates).
From: Gagliardi et al. How can we improve guideline use? A conceptual framework of implementability. Implementation Science 2011, 6:26.

## Accounting for comorbidities when developing guidelines

An emerging topic of interest when developing practice guidelines is how to shape guideline development to account for patients who have multiple medical conditions. This issue was highlighted by Boyd and colleagues, who assessed the applicability of guidelines to a hypothetical 79 year old woman with five chronic conditions: osteoporosis, osteoarthritis, diabetes, hypertension, and chronic obstructive pulmonary disease [20]. Boyd and colleagues noted that guidelines for these conditions, with the exception of diabetes, did not discuss recommendations for management in patients with other chronic conditions. Applying the guidelines to their hypothetical patient, they found the patient would need to be advised of seven self-management tasks, the clinician would be responsible for performing 18 tasks, the patient would be recommended to take 12 separate medications in 19 doses per day, and that certain medications recommended for one condition could exacerbate symptoms or interact with medications for other conditions (e.g., NSAIDs for osteoarthritis potentially raising the blood pressure in hypertension; hydrochlorothiazide for hypertension potentially raising glucose levels in diabetes). Boyd and colleagues concluded that attempting to apply current guidelines to patients with multiple health conditions may have undesirable effects.

It is perhaps not surprising that guidelines have remained largely silent about what to do when patients have multiple conditions and that there has been little progress in rectifying this situation. Clinical practice guidelines aspire to be evidence-based, and often the studies used as the evidentiary base have excluded patients with multiple chronic conditions. Consequently, there rarely are data about the effect that the presence of other health conditions have on the treatments and outcomes of care for a particular condition - knowledge that would translate into differences in treatment recommendations in a guideline depending on the presence of certain other chronic conditions. The exception to this general rule is diabetes, where there are data about differences in outcomes for patients with hypertension and with cardiovascular disease, in terms of optimal blood pressure control and LDL levels, and these data have been translated into differential treatment recommendations in practice guidelines. Having epidemiologic data on the frequency of co-morbidities with the target condition would inform the discussion regarding the presence or absence of evidence about how these comorbidities should or should not influence treatment decisions. In the context of diabetes, Piette and Kerr have articulated the concept of “concordant” versus “discordant” comorbidities, with “concordant” comorbidities being ones that tend to have similar management plans (for example, the triad of hypertension, diabetes and coronary artery disease) while discordant comorbidities do not have the feature and do not share any underlying predisposing factor (diabetes and asthma or diabetes and prostate cancer) [21]. This concept may be useful to guideline development committees when thinking about dealing with recommendations for patients with co-morbidities. Likewise, Boyd and colleagues have developed a framework for thinking about disease severity in older adults, which includes the effect of the interaction of disease severity on other diseases [22]. Since treating one condition optimally (such as use of increased doses of non-steroidal anti-inflammatory drugs for osteoarthritis) may cause an increased risk of side effects (gastrointestinal bleeding) that can be mitigated by adding another medication (proton pump inhibitors) that unfortunately has a possible deleterious action on the outcomes of another health condition (the use of clopidogrel in patients who have had coronary artery revascularization interventions), weighing the risks and benefits of treatments across a patient’s health conditions will be necessary, and will likely involve a discussion with the patient about preferences for outcomes. Methods to incorporate individual patient values is an active area of research [23]. From the clinical area of respiratory disease practical guidance will be forthcoming (Fabbri LF, BoydC, Boschetto P, Rabe KF, Buist AS, Yawn B, Leff B, Kent DM, Schünemann HJ. How to integrate multiple comorbidities in guideline development: An official ATS/ERS Workshop Report. Forthcoming).

## Summary

In this third and final paper in the series we discussed the issues around: reviewing, reporting, and publishing guidelines; updating guidelines, and the emerging issues of enhancing implementability and how guidelines approach dealing with patients with co-morbid conditions.

## Competing interests

MPE is Editor in Chief of Implementation Science; Jeremy Grimshaw is an Editorial Board member. All decisions on this paper were made by another editor. The authors have all been involved in guideline development for a range of different organizations.

## Authors' contributions

All authors contributed to the writing of this article and approved the final draft.
